# A Minireview of the Promising Drugs and Vaccines in Pipeline for the Treatment of COVID-19 and Current Update on Clinical Trials

**DOI:** 10.3389/fmolb.2021.637378

**Published:** 2021-06-09

**Authors:** Jeyanthi Venkadapathi, Venkat Kumar Govindarajan, Saravanan Sekaran, Santhi Venkatapathy

**Affiliations:** ^1^ Department of Biotechnology, SRM Arts and Science College, Chengalpattu, India; ^2^ Department of Biotechnology, Ponnaiyah Ramajayam Institute of Science and Technology, Thanjavur, India; ^3^ Department of Biotechnology, School of Chemical and Biotechnology, SASTRA Deemed University, Thanjavur, India; ^4^ Department of Anatomy, SRM Medical College Hospital and Research Centre, SRM Institute of Science and Technology, Chengalpattu, India

**Keywords:** COVID-19, antiviral drugs, vaccines, clinical trial, update

## Abstract

The COVID-19 is affecting thousands of peoples day by day and continues to spread across the world. The present review has focused on promising repurposing drugs, including remdesivir, lopinvar/retinovar, favipiravir, hydroxychloroquine, monoclonal antibodies and vaccines against the SARS-CoV-2 infection. Besides, our review has also focused on many organizations that are in the race to develop vaccines using various approaches including DNA, RNA, viral vectors and subunit proteins against this highly contagious respiratory disease. The spike protein is being studied by scientists all over the world to develop potential vaccines. The antiviral drugs, antibodies and vaccines developed by various researchers around the world have entered clinical trials in humans. The current clinical trials for antiviral agents and vaccines with promising outcomes are being discussed. So far, four vaccines developed by the Pfizer-BioNTech vaccine, the Johnson and Johnson vaccine and two AstraZeneca vaccines (produced by SKBio in the Republic of Korea and Serum Institute of India) are approved by the World Health Organization for public use.

## Introduction

The current outbreak of COVID-19 (Coronavirus disease-19) has created a major health problem worldwide. The respiratory tract infection caused by the novel coronavirus strain known as Severe Acute Respiratory Syndrome Coronavirus 2 (SARS-CoV-2), which was first identified in Wuhan, China at the end of 2019 and quickly spread across the world within a short period. (Shereen et al., 2020). As of april 11, 2021, the virus has killed 2, 941, 533 people around the world (CSSE, 2021). The World Health Organization (WHO) on March 11, 2020, has declared the SARS-CoV-2 outbreak a global pandemic. Due to the lack of successful vaccine candidates or antiviral molecules, the infection and mortality rate have increased globally (Umesh and Yadav, 2021). The WHO, European Medicines Agency (EMA), United States Food and Drug Administration (FDA), the Chinese Government and drug manufacturers have collaborated with various academic and industry researchers to improve the development of vaccines, antiviral drugs, and post-infection therapies. The most important target proteins for SARS-CoV-2 include papain-like protease, RNA-dependent RNA polymerase, helicase, S protein, and ADP-ribose diphosphatase (Venkat Kumar et al., 2020).

Currently, few clinically approved repurposed antiviral drugs such as favipiravir, remdesivir, lopinavir, hydroxychloroquine (or chloroquine) and dexamethasone was targeted against SARS-CoV-2 (Jeyanthi and Kumar, 2020). In april 2020, these antiviral drugs, monoclonal antibodies, and few vaccine candidates have entered the human clinical trials. Table 1 represents the list of repurposed drugs and updates on a clinical trial. At present, few efficient antiviral agents are under clinical trial to fight the disease and the clinical aspects of those agents are explored. In the present study, we highlighted some medications to find an effective treatment for this deadly virus.

**TABLE 1:** Recently approved repurposed drugs for COVID-19 treatment.

Sl. No	Drug	Developer/ company	Original use	Molecules/Enzyme inhibition	Clinical trial status
1.	Danoprevir combination with Ritonavir	Roche Pharma, Switzerland	Hepatitis C virus (HCV)	NS3/4A protease inhibitor	Phase IV
2.	Umifenovir	JSC Pharmstandard, Russia	Influenza	Inhibits target cell membrane fusion with virus.	Phase IV
3	Hydroxy-chloroquine	Ipca Laboratories, Zydus Cadila and Wallace Pharmaceuticals, India	Malaria, Rheumatoid arthritis, Chronic discoid lupus erythematosus and Systemic lupus erythematosus	Terminal glycosylation of ACE2	Phase III/IV (Discontinued by WHO on July 2020)
4.	Ramdesivir	Gilead Sciences, United States	Ebola and Nipah virus	RNA-dependent RNA polymerase	Phase III/IV
5.	Lopinavir/Ritonavir	Abbott Laboratories, United States	Human immunodeficiency viruses (HIV)	Protease	Phase III/IV
6	Favipiravir	Fujifilm Toyama Chemical company Limited, Japan	Influenza virus	RNA-dependent RNA polymerase	Phase III/IV
7.	Dexamethasone	Zydus Cadila pharmaceutical company, India	Rheumatic problems, asthma, skin and lung diseases	Phospholipase A2	Phase III
8.	Oseltamivir	Taj pharmaceutucals, India	Influenza	Neuraminidase inhibitor	Phase III
9.	Sarilumab	Regeneron Pharmaceuticals, United States and Sanofi pharmaceutical company, France	Rheumatoid arthritis	anti IL-6 receptor monoclonal antibody	Phase III
10.	Tocilizumab	Genentech, United States and Hoffmann-La Roche, Switzerland	Rheumatoid arthritis	anti IL-6 receptor monoclonal antibody	Phase III
11.	Lezilumab	Humanigen, United States	New monoclonal antibody against pneumonia	anti GM-CSF receptor	Phase III

## Antiviral Drugs

### Remdesivir

Remdesivir is a broad-spectrum antiviral drug designed by Gilead Sciences, an American biopharmaceutical company. Previously, this drug has proven effective *in vitro* antiviral activity against Ebola, Nipah, and Respiratory syncytial virus. Subsequently, the drug was shown to be effective against other coronaviruses such as SARS (Severe acute respiratory syndrome) and MERS (Middle East respiratory syndrome) both *in vitro* and in animal models (Scavone et al., 2020). The SARS CoV-2 virus replication takes place using a particular enzyme known as the RNA-dependent RNA polymerase. Researchers proved that the remdesivir could block this enzyme necessary for viral replication (Gordon et al., 2020). Remdesivir is an adenosine nucleoside analog, which incorporates into viral RNA chains, causing premature breakage by interfering with viral replication. Many clinical trials were underway assessing remdesivir as a potential treatment for COVID-19 treatment. In China, during the period of February-March 2020, a clinical trial on remdesivir showed ineffective treatment of COVID-19 patients and caused many harmful effects (Wang et al., 2020). In March 2020, scientists proved that the progression of COVID-19 was reduced in rhesus macaque monkeys after treatment with remdesivir (Williamson et al., 2020). On April 29, 2020, the United States National Institute of Allergy and Infectious Diseases (NIAID) reported that the drug provided a 31% faster recovery in 11 days. On the other hand, a double-blind, randomized, placebo-controlled trial was carried out by the United States National Institutes of Health. Patients were randomly assigned to receive either remdesivir (200 mg loading dose on day 1, followed by up to nine more days of 100 mg daily) or placebo for up to 10 days of treatment. The study suggested that remdesivir was found to be effective in COVID-19 patients and reduced the recovery time from 15 to 11 days. In August 2020, the FDA expanded the emergency use authorization (EUA) for the use of remdesivir in treating COVID-19 patients. Thereafter, on October 22, 2020, FDA approved and also revised the EUA to authorize the use of this drug.

### Favipiravir

Favipiravir is an antiviral drug used to treat the influenza virus. The drug has also shown antiviral activities against several RNA viruses. Hence, it could be a promising agent for SARS-CoV-2 infection, which is also an RNA virus (Dong et al., 2020). Favipiravir is metabolized to its active form favipiravir-ribofuranosyl-5′-triphosphate (favipiravir-RTP), which inhibits RNA-dependent RNA polymerase (RdRp) enzyme. It interferes with the elongation of the RNA strand and prevents viral replication (Furuta et al., 2009). In China, a clinical trial on favipiravir was initiated in February 2020. The test was conducted on 80 patients by the National Clinical Research Center for Infectious Diseases. The potent antiviral action was noticed with fewer adverse effects. CT (computerized tomography) scan results revealed the eradication of the virus in 91% of people (Cai et al., 2020; Dong et al., 2020). Later, in Wuhan, the research was carried out in 240 COVID-19 patients and the scientists observed that the patients treated with favipiravir have recovered from cough and fever, but no changes were observed in patients receiving prolonged ventilation (Regalado, 2020). On March 22, 2020, Italy approved this drug for clinical trials only in the severely affected COVID-19 patients. In India, the drug was approved for treatment under the name “Fabiflu”.

### Lopinavir/Ritonavir

The combination drug lopinavir/ritonavir (Kaletra) belongs to the class of protease inhibitors used for the treatment of the human immunodeficiency virus (HIV). The drug inhibits the replication of the virus by binding to the HIV protease enzyme. Some researchers have proved the efficacy of the drug against other coronaviruses such as SARS and MERS. Cao et al. (2020) conducted a trial of Lopinavir–Ritonavir in adults Hospitalized with severe COVID-19. Their study result showed that the combinational drug was not effective for the treatment. However, the WHO included the drug in the “Global Solidarity trial” for the treatment. Currently, this drug is under clinical trial for the treatment of COVID-19 positive patients with cancer and immune suppression (National Library of Medicine (U.S.), 2020-2021c).

### Hydroxychloroquine

It is generally used to treat malaria, rheumatoid arthritis, systemic lupus erythematosus, and porphyria cutanea tarda. In the malarial parasites, hydroxychloroquine accumulate in the lysosomes, increase the pH of the vacuole, inhibits the ability of parasites to proteolyze the hemoglobin and prevents the growth of the parasite (Lei et al., 2020). Likewise, in human cells, the drug increased the pH in endosomes and prevents the entry of SARS-CoV-2 virus particles. The Angiotensin-converting enzyme 2 (ACE2) enzymes are normally expressed at the outer surface of human cells. ACE2 serves as the functional receptor for the entry of SARS-CoV-2 (Venkat Kumar et al., 2020). The terminal glycosylation of ACE2 is inhibited by hydroxychloroquine, prevents the interaction of ACE2 with SARS-CoV-2 “spike” protein, and hence inhibits the entry of the virus. Clinical studies from China showed that the hydroxychloroquine reduced the risk of progression to severe illness in COVID-19 patients (Chen et al., 2020). In February 2020, a non-randomized study in a small sample size from France shows that the hydroxychloroquine plus azithromycin treatment reduced the viral load in COVID-19 patients (Gautret et al., 2020). Another study from France reported that the hydroxychloroquine plus azithromycin have no strong antiviral activity in severely affected COVID-19 patients (Molina et al., 2020). However, in april 2020, Hydroxychloroquine was approved by FDA for emergency use. Based on the clinical research analysis and scientific data, the FDA reported that hydroxychloroquine is ineffective in treating COVID-19 and revoked the emergency use authorization in June 2020.

## Monoclonal Antibodies

### Sarilumab

Sarilumab is a human monoclonal antibody generally used for the treatment of rheumatoid arthritis in adults. Zhao (2020) suggested that the sarilumab antibody could inhibit the production of a cytokine IL-6 in the patients with COVID-19 pneumonia. A randomized, double-blind, placebo-controlled, phase three trial of sarilumab in patients admitted to Hospital with severe or critical COVID-19 was studied. Their result shows that the sarilumab efficacy is poor in patients admitted to Hospital with COVID-19 and receiving supplemental oxygen (Lescure et al., 2021).

### Tocilizumab

Tocilizumab is another monoclonal antibody against IL-6 used for the treatment of rheumatoid arthritis and systemic juvenile idiopathic arthritis in children. The drug could prevent the expression of IL-6 in COVID-19 patients (Luo et al., 2020). In March 2021, FDA approved this tocilizumab for clinical trials to evaluate its safety and efficiency (National Library of Medicine, 2021). But, the scientific evidence showed that a randomized double-blind clinical trial was not effective to prevent death in severely affected COVID-19 patients.

### Lenzilumab

Lenzilumab is a monoclonal antibody used for the treatment of chronic and juvenile myelomonocytic leukemia. Lenzilumab is used to block the expression of the granulocyte-macrophage colony-stimulating factor (GM-CSF). The COVID-19 Hospitalized patients have higher levels of the inflammatory cytokine GM-CSF in the plasma, which is reported to be a key to trigger the disease (Huang et al., 2020). In May 2020, FDA has approved lenzilumab to enter the clinical trial (National Library of Medicine (U.S.), 2020-2021b).

## Vaccines

Over the past century, numerous successful attempts have been made to develop vaccines for polio, cholera, measles, typhoid, and tetanus. Apart from attenuated vaccines, conjugate and subunit vaccines are also proved to be efficient against pneumonia, sepsis, and meningitis (Kim et al., 2020). Currently, more than 150 vaccine candidates for SARS-CoV-2 are under development at various stages. There has been an increased focus on the pre-clinical development of COVID-19 vaccines by many research institutes and vaccine manufacturers around the world. Currently, the predominant vaccine platforms for pre-clinical studies included are DNA, RNA, inactivated virus, viral vector (Replicating and Non-Replicating), live attenuated virus, protein subunit and virus-like particle (VLP) (Bezbaruah et al., 2021; Borah et al., 2021). Though many vaccines have been included in clinical trials, vaccine candidates such as AZD1222 (Covishield, Vaxzevria), BNT162b2 mRNA-1273 and CoronaVac are in the phase IV clinical trial (Table 2). Currently (as of May 11, 2021), over 99 vaccine candidates are under assessment in clinical trials on humans and 184 under pre-clinical trials on animals (WHO, 2020). Recently, regulatory authorities in some countries have authorized mRNA vaccines, recombinant adenoviral vectors vaccines, and commonly used inactivated virus vaccines for emergency use. So far, globally, there have been 30 active vaccine projects that involve the development of mRNA vaccines, recombinant adenoviral vectors vaccines, and inactivated virus vaccines. Despite the number of vaccine development projects, WHO has so far validated only six vaccines globally, the Pfizer-BioNTech vaccine, the Johnson and Johnson vaccine, Sputnik V, Sinopharm-BIBP, Moderna and two AstraZeneca vaccines (produced by SKBio in the Republic of Korea and Serum Institute of India) for emergency use. These vaccines are proved to be safe and effective by the WHO Strategic Advisory Group of Experts on Immunization (Acharya et al., 2021). The Pfizer-BioNTech vaccine is a mRNA type vaccine that can be delivered intramuscular with two dose series separated by a 21 days interval. Based on the published evidence from clinical trials, the Pfizer-BioNTech vaccine is reported to be 95% effective at preventing laboratory-confirmed COVID-19 illness in people without the history of previous infection (Centers for Disease Control and Prevention, 2021b). The Johnson and Johnson vaccine is however a viral vector type which can be delivered intramuscular with a single dose. Based on the clinical trial publication evidences, the Johnson and Johnson vaccine is reported to be 66.3% effective after 2 weeks of vaccination at preventing laboratory-confirmed COVID-19 illness in people who had no history of prior infection (Centers for Disease Control and Prevention, 2021a). Particularly, AstraZeneca vaccine is co-invented by the University of Oxford and its spin-out company, Vaccitech. It is a weakened version of a common cold virus (adenovirus) that causes infections in chimpanzees and contains the genetic material of the SARS-CoV-2 virus spike protein. The vaccine is delivered intramuscular with a two dose series that are separated by 4 weeks. The AstraZeneca vaccine phase III clinical trial data is reported to be 79% effective at preventing symptomatic COVID-19 and 100% efficacy at preventing severe disease and Hospitalization (National Library of Medicine (U.S.), 2020-2021a). The Moderna COVID-19 vaccine is developed by Moderna, the United States National Institute of Allergy and Infectious Diseases (NIAID) and the Biomedical Advanced Research and Development Authority (BARDA). It is a mRNA-1273 vaccine encapsulated with lipid nanoparticles, delivered intramuscular with a two dose series separated by 28 days. The phase III clinical trial reported to be 94.1% efficacy at preventing COVID-19 illness, including severe disease (Baden et al., 2021). Sinopharm BIBP is a COVID-19 vaccine produced by the China National Pharmaceutical Group (Sinopharm) and its Beijing Institute of Biological Products (BIBP). It is an inactivated virus vaccine, delivered intramuscular with a two dose series separated by 21 days. The phase III clinical trial reported to be 79% efficacy against COVID-19 symptomatic and Hospitalized patients (WHO, 2021). Covaxin is India’s first vaccine, developed by Bharat Biotech Company in collaboration with the Indian Council of Medical Research (ICMR) and National Institute of Virology (NIV). The vaccine is an inactivated SARS-CoV-2 virus, delivered intramuscular with a two dose series separated by 28 days. Covaxin phase III clinical trial data is reported to be 81% interim efficacy in preventing COVID-19 illness in people without prior infection after the second dose (National Library of Medicine (U.S.), 2020-2021d). Russia’s first approved COVID-19 vaccine is Sputnik V. It is an adenoviral DNA-based vaccine. The phase III trial of Sputnik V is reported to be 91.6% efficacy against COVID-19 illness patients (Jones and Roy, 2021). The vaccines currently in clinical trials are summarized in Table 2. The cold storage of vaccines is crucial to reduce the loss of stability, and immunogenicity. The two promising COVID-19 mRNA vaccines, including BioNTech/Pfizer and Moderna are to be stored in ultra-cold storage conditions at −70°C and −18°C, respectively. These vaccines are encapsulated in lipid nanoparticles (LNPs) which have the advantage of delivering the mRNA by protecting them from enzyme degradation and it can effectively deliver mRNA vaccines into the cell cytosol through a series of endocytosis mechanisms. (Acharya et al., 2021; Baden et al., 2021). The ultra-cold storage of the BioNTech/Pfizer vaccine would be unsuitable for low and middle-income countries due to a shortage of cold-chain infrastructure. The maintenance of mRNA-LNPs in a frozen form will be a major challenge for transport, storage, and distribution in developing countries, resulting in reduced immunization rates. On the other hand, AstraZeneca’s vaccine can be stored, transported, and handled at normal refrigerated conditions (2–8 °C) for at least six months. Moreover, comparing to the other types of vaccine, the cost of the AstraZeneca’s vaccine is very less (around $2-4 per dose). Hence, it can be easily produced for a larger population and distributed using existing medical facilities in developing countries.

**TABLE 2:** Vaccine candidates under investigation in the clinical trial of COVID-19.

Sl. No	Vaccine candidate	Description/type	Manufacturer/Institution and location	Trial phase
1	AZD1222 (Covishield, Vaxzevria)	adenoviral vector	AstraZeneca, University of Oxford and SK bioscience South Korea	Phase IV
2	CoronaVac	Inactivated or killed SARS-CoV-2	Sinovac Biotech, China	Phase IV
3	BNT162b2	RNA	Pfizer, United States, BioNTech, Germany and Fosun Pharma, China and	Phase IV
4	mRNA-1273	Lipid nanoparticle assisted mRNA delivery	Moderna and United States National Institute of Allergy and Infectious Diseases, United States	Phase IV
5	Ad5-nCoV	Recombinant adenoviral vector	CanSino Biologics, China	Phase III
6	NVX-CoV2373	SARS-CoV-2 recombinant spike protein nanoparticles	Novavax, Australia	Phase III
7	BBIBP-CorV (Sinopharm)	Inactivated SARS-CoV-2	Beijing Institute of Biological Products, China	Phase III
8	Minhai COVID-19 vaccine	Inactivated SARS-CoV-2	Minhai Biotechnology Co., China	Phase III
9	Sputnik V	Adenoviral vector	Gamaleya Research Institute, Russia	Phase III
10	Ad26.COV2.S	Adenoviral vector	Janssen Pharmaceutical Companies, Belgium	Phase III
11	Inactivated	Inactivated SARS-CoV-2	Chinese Academy of Medical Sciences, China	Phase III
12	ZF2001 (RBD-Dimer)	Protein Subunit	Anhui Zhifei Longcom Biopharmaceutical/Institute of Microbiology, Chinese Academy of Sciences, China	Phase III
13	CVnCoV	RNA	Curevac, biopharmaceutical company, Germany	Phase III
14	CoviVac	Inactivated SARS-CoV-2	Chumakov Center, Russian Academy of Sciences	Phase III
15	CIGB-66 (ABDALA)	Protein subunit	Center for Genetic Engineering and Biotechnology, Cuba	Phase III
16	ZyCoV-D	DNA	Zydus Cadila, India	Phase III
17	BBV152 (Covaxin)	Inactivated SARS-CoV-2	Bharat Biotech, India	Phase III
18	EpiVacCorona	Peptide subunit	State Research Center of Virology and Biotechnology VECTOR, Russia	Phase III
19	GRAd-COV2	Adenovirus vector	Lazzaro Spallanzani National Institute for Infectious Diseases, Italy	Phase II/III
20	COVIran Barakat	Inactivated SARS-CoV-2	Barakat Pharmaceutical Group, Iran	Phase II/III
21	INO-4800	Electroporation delivered DNA vaccines	Inovio Pharmaceuticals, United States and International Vaccine Institute, South Korea	Phase II/III
22	AG0302-COVID-19	DNA Vaccine (plasmid)	Osaka University/ AnGes/ Takara Bio Inc., Japan	Phase II/III
23	SCB-2019	Protein Subunit	Clover Biopharmaceuticals, china	Phase II/III
24	UB-612	Protein Subunit	United Biomedical Inc., United States and DASA, Brazil	Phase II/III
25	CoVLP	Virus like particles	Medicago Inc., Canada	Phase II/III
26	MVC-COV1901	Protein Subunit	Medigen Vaccine Biologics Corporation, Taiwan	Phase II
27	Nanocovax	SARS-CoV-2 recombinant spike protein subunit	Nanogen Pharmaceutical Biotechnology JSC, Vietnam	Phase II
28	ERUCOV-VAC	Inactivated SARS-CoV-2	Health Institutes of Turkey	Phase II
29	DelNS1-2019-nCoV-RBD-OPT	Replicating Viral Vector	Beijing Wantai Biological Pharmacy, China	Phase II
30	ARCT-021	RNA	Arcturus Therapeutics, United states and Duke–NUS Medical School, Singapore	Phase II
31	LV-SMENP-DC	Dendritic cells modified with lentiviral vector	Shenzhen Geno-Immune Medical Institute, China	Phase I/II
32	GX-19	DNA Vaccine	Genexine Consortium, South Korea	Phase I/II
33	KBP-201 (RBD-based)	Protein Subunit	Kentucky Bioprocessing, Inc., United States	Phase I/II
34	IIBR-100 (Brilife)	Vesicular stomatitis vector (recombinant)	Israel Institute for Biological Research	Phase I/II
35	RBD SARS-CoV-2 HBsAg VLPs	Virus-like particle	Serum Institute of India Accelagen Pty, Australia and SpyBiotech, United kingdom	Phase I/II
36	GBP510	Protein subunit	SK Bioscience Co., Ltd. London and CEPI, Norway	Phase I/II
37	VBI-2902	Virus-like particle	Variation Biotechnologies, United States	Phase I/II
38	NDV-HXP-S	Viral vector	Mahidol University, Thailand	Phase I/II
39	EuCorVac-19	Protein subunit	EuBiologics Co, South Korea	Phase I/II
40	AV-COVID-19	Viral vector	AIVITA Biomedical, Inc., United States and Ministry of Health, Indonesia	Phase I/II
41	COVID-eVax	DNA	Takis Biotech, Italy	Phase I/II
42	ChulaCov19	RNA	Chulalongkorn University, Thailand	Phase I/II
43	Bio E COVID-19 (BECOV2D)	Subunit (antigen)	Biological E. Limited, India and Baylor College of Medicine, United States	Phase I/II
44	Covigenix VAX-001	DNA	Entos Pharmaceuticals Inc., Canada	Phase I
45	bacTRL-Spike	DNA	Symvivo Corporation, (biotechnology company) Canada	Phase I
46	Covid-19/aAPC	Lentiviral vector	Shenzhen Geno-Immune Medical Institute, China	Phase I
47	MVA-SARS-2-S	Non-Replicating Viral Vector	University of Munich (Ludwig-Maximilians), Germany	Phase I
48	COVAX-19	Protein Subunit	Vaxine Pty Ltd/Medytox, Australia	Phase I
49.	Molecular clamp stabilized Spike protein with MF59 adjuvant	Protein Subunit	University of Queensland/CSL Ltd., Australia	Phase I
50	CoVac-1	Protein Subunit	University Hospital Tuebingen, Germany	Phase I
51	COVI-VAC	Attenuated	Codagenix Inc., United states and Serum institute of India	Phase I
52	PTX-COVID19-B	RNA	Providence Therapeutics, Canada	Phase I
53	COVIGEN	DNA	University of Sydney, Australia	Phase I
54	BBV154	Adenovirus vector	Bharat Biotech, India	Phase I
55	NBP2001	DNA	SK Bioscience Co. Ltd., South Korea	Phase I
56	DelNS1-nCoV-RBD LAIV	Attenuated	University of Hong Kong	Phase I
57	LNP-nCoVsaRNA	RNA	Imperial College London	Phase I
58	ChulaCov19 mRNA vaccine	RNA	Chulalongkorn University, Thailand	Phase I
59	AdCOVID	Non-replicating Viral vector	Altimmune, Inc., United States	Phase I
60	mRNA-1283	RNA	Moderna Inc., United States	Phase I

Source: https:// www.who.int/publications/m/item/draft-landscape-of-covid-19-candidate vaccines.

## Conclusion

In the present study, the possible therapeutic options described are solely based on the latest research findings for the treatment of COVID 19. We have summarized the current status of the repurposing drugs, including remdesivir, favipiravir, lopinvar/retinovar, hydroxychloroquine, monoclonal antibodies and vaccines against the SARS-CoV-2 infection. The development of new drugs is a complex and prolonged process. Hence, repurposed drugs could be an alternative to combat COVID-19. However, vaccines under clinical trials are showing great results compared to the other therapeutics options. More than 100 vaccines are under study, among that only four vaccines have been approved by WHO for the prevention and treatment of COVID-19. The WHO encourages COVID-19 vaccine manufacturers in many countries and ensures its safety in immunization. The vaccines were found to be safe for adults, including those with pre-existing auto-immune disorders. Several vaccines have received emergency use authorization in many countries but careful monitoring in high-risk individuals over the age of 60 is still required.
